# From the Unexpected: Unveiling the Diverse Presentations of Extraosseous Ewing's Sarcoma

**DOI:** 10.7759/cureus.89114

**Published:** 2025-07-31

**Authors:** Akanksha Giri, Rajoo Ramachandran, Kiran Gopinathan, Pradosh Kumar Sarangi

**Affiliations:** 1 Radiology, Sri Ramachandra Institute of Higher Education and Research, Chennai, IND; 2 Radiology, All India Institute of Medical Sciences, Deoghar, Deoghar, IND

**Keywords:** cd99, diagnostic delay, extraosseous ewing's sarcoma, immunohistochemistry, mediastinal mass, mesenteric tumor, nkx2.2, small round blue cell tumor, vdc-ie chemotherapy

## Abstract

Extraosseous Ewing's sarcoma (EES) is a rare but aggressive soft tissue malignancy of small round blue cells and commonly poses a diagnostic challenge because of its nonspecific anatomical locations and association with other neoplasms. This series of cases presents five uncommon presentations of EES in adult patients in the form of tumors present in the mediastinum, mesentery, right suprarenal gland, infraclavicular soft tissue, and prostate. The clinical presentations varied from rapidly worsening respiratory failure caused by a mediastinal tumor to a preoperatively misdiagnosed gastrointestinal stromal tumor that was subsequently diagnosed as EES post-surgery. Immunohistochemistry (IHC) was crucial in diagnosis, and all the cases were strongly positive for CD99 with supporting markers such as NKX2.2 and FLI-1. Multimodal treatment with chemotherapy (vincristine, doxorubicin (adriamycin), cyclophosphamide, ifosfamide, and etoposide (VDC-IE) regimen), surgery, and radiotherapy was used, and results were variable, with one patient dying of neutropenic sepsis and others having a favorable response to therapy with follow-up. This series puts forward the significance of keeping EES in the differential diagnosis of adult deep-seated or visceral soft tissue masses and the imperative need for prompt histopathological and immunohistochemical correlation to aid proper management.

## Introduction

Extraosseous Ewing's sarcoma (EES) is a rare and extremely aggressive malignancy within the Ewing sarcoma family of tumors (ESFT), distinguished by its occurrence outside of the skeletal system. Initially reported by James Ewing in 1921 as a diffuse endothelioma of bone, the identification of Ewing's sarcoma as extraosseous in location has subsequently broadened our knowledge of this tumor’s biology and clinical variability. Although having principal molecular hallmarks in common with osseous Ewing's sarcoma, particularly the pathognomonic EWSR1-FLI-1 gene produced by the t(11;22)(q24;q12) translocation, EES presents special diagnostic and therapeutic challenges because of its extensive anatomical spread and variable clinicopathological features [1]. The ability of the tumor to occur in almost any soft tissue site, such as the trunk, extremities, retroperitoneum, paravertebral areas, and even unusual sites such as the central nervous system, skin, and visceral organs, tends to result in a delay or misdiagnosis, especially in adults where it is not so anticipated [2].

The clinical presentation of EES is as varied as its anatomical distribution, from aggressive, growing, painful tumors to incidental detection in imaging studies, further clouding early diagnosis [3]. In contrast with osseous Ewing's sarcoma, which is more common in children and adolescents, EES has a wider age predilection, with cases reported in both the pediatric and adult age groups, at times simulating other soft tissue sarcomas, carcinomas, or even benign tumors [4]. This diagnostic uncertainty requires a high level of suspicion, especially in young adults with soft tissue masses, and highlights the irreplaceable position of immunohistochemistry (IHC) and molecular genetic analysis in diagnosing this condition. Histologically, EES usually shows small round blue cell morphology, with intense CD99 (MIC2) membranous labeling, although the differential diagnosis involves other small round cell tumors such as lymphoma, rhabdomyosarcoma, and small cell carcinoma, making a wide-ranging diagnostic workup mandatory.

Apart from its diagnostic challenges, EES is infamous for its aggressive biologic behavior, including a high local recurrence rate and early hematogenous metastasis, often to the lungs, bone marrow, and other soft tissues. EES has traditionally been thought to have a poorer prognosis compared to skeletal Ewing's sarcoma, although current advances in multimodal therapy, combining neoadjuvant chemotherapy, wide surgical resection, and radiotherapy, have markedly enhanced survival results [5]. The present treatment of choice adheres to guidelines set for osseous Ewing's sarcoma and employs vincristine, doxorubicin (adriamycin), cyclophosphamide, ifosfamide, and etoposide (VDC-IE) as the mainstay, although the best forms of treatment for EES are the subject of continued investigation because of its rarity and biologic heterogeneity [6]. New therapeutic strategies, such as targeted therapies against the EWSR1-FLI-1 fusion protein, immune checkpoint inhibitors, and adoptive T-cell therapies, have the potential for refractory or metastatic disease, although their effectiveness in EES per se needs further study.

Due to the rarity and clinical variability of the tumor, accurate diagnosis, staging, and treatment planning require multidisciplinary collaboration between oncologists, pathologists, radiologists, and surgeons. Large-scale genomic studies and international registries would also be helpful to further characterize the molecular drivers, prognostic factors, and best therapeutic approaches for EES [7,8]. This case series is intended to give an overall picture of the clinicopathological range of, and diagnostic issues with, EES, with a focus on the early recognition, molecular diagnosis, and tailored treatment modalities as a measure of enhancing patient outcome.

## Case presentation

Case 1: Recurrent mediastinal extraosseous Ewing's sarcoma with cardiopulmonary compromise

A 32-year-old female patient came with complaints of worsening shortness of breath, cough with expectoration, and generalized weakness for four days. She had a history of superior vena cava (SVC) thrombosis in 2022, which prompted further evaluation that turned out to reveal a mediastinal mass. The mass was resected on March 26, 2022, and histopathological examination revealed sheets of small round blue cells. Immunohistochemistry (IHC) established the diagnosis of extraosseous Ewing's sarcoma (EES) to be CD99 and NKX2.2 positive. She received systemic chemotherapy with VAC regimen (vincristine, adriamycin, and cyclophosphamide), followed by etoposide and ifosfamide.

**Figure 1:**
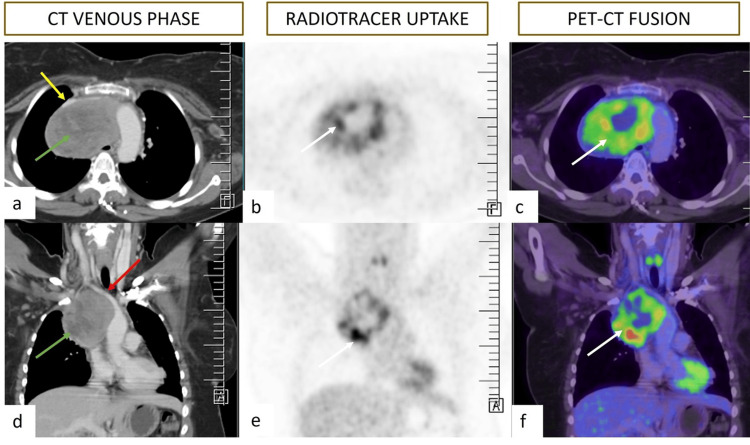
Axial (a, b, and c) and coronal (d, e, and f) reformatted PET-CT images show an FDG-avid heterogeneous mass lesion (SUVmax: 6.7) in the superior mediastinum, anterior and right to the trachea. Non-enhancing necrotic areas are seen within (green arrow). The superior vena cava is compressed and pushed anteriorly (yellow arrow). The right brachiocephalic trunk is pushed superiorly (red arrow). PET-CT: positron emission tomography-computed tomography, FDG: fluorodeoxyglucose, SUVmax: maximum standardized uptake value

In 2023, the patient came back with New York Heart Association (NYHA) class IV dyspnea and cough. Positron emission tomography-computed tomography (PET-CT) imaging showed a large, fluorodeoxyglucose (FDG)-avid heterogeneously enhancing mass lesion in the superior mediastinum, measuring around 6.7 maximum standardized uptake value (SUVmax) (Figure 1). The lesion was invading the SVC and surrounding mediastinal structures such as the right auricle, right pulmonary artery, and right main bronchus. Extensions of the mass were also seen into the retroperitoneal compartment. The persistent tumor was invading the right pleura, pericardial fat pad, and liver subcapsular spaces, suggesting metastatic disease.

On presentation, the patient presented with a massive right-sided pleural effusion and reduced air entry on the right side. Echocardiogram revealed global hypokinesia and moderate left ventricular impairment with an ejection fraction of 38%. Due to the massive effusion, a pulmonology referral was requested, and pleurocentesis was done, which gave an exudative effusion. Effusion, however, recurred, and an intercostal drain (ICD) was inserted after a consultation with cardiothoracic surgery. Even after these interventions, the respiratory distress of the patient deteriorated. She was counselled for further palliative systemic chemotherapy but chose to remain at a different facility for further care. She was discharged on an ICD tube in place and referred for oncology services for ongoing management. This case points out the aggressive progression of EES, its propensity for recurrence, and the need for a multidisciplinary team approach to manage mediastinal masses with cardiopulmonary extension.

Case 2: Septic neutropenia due to chemotherapy for a tumor misdiagnosed as gastrointestinal stromal tumor (GIST) and subsequently confirmed to be EES

**Figure 2:**
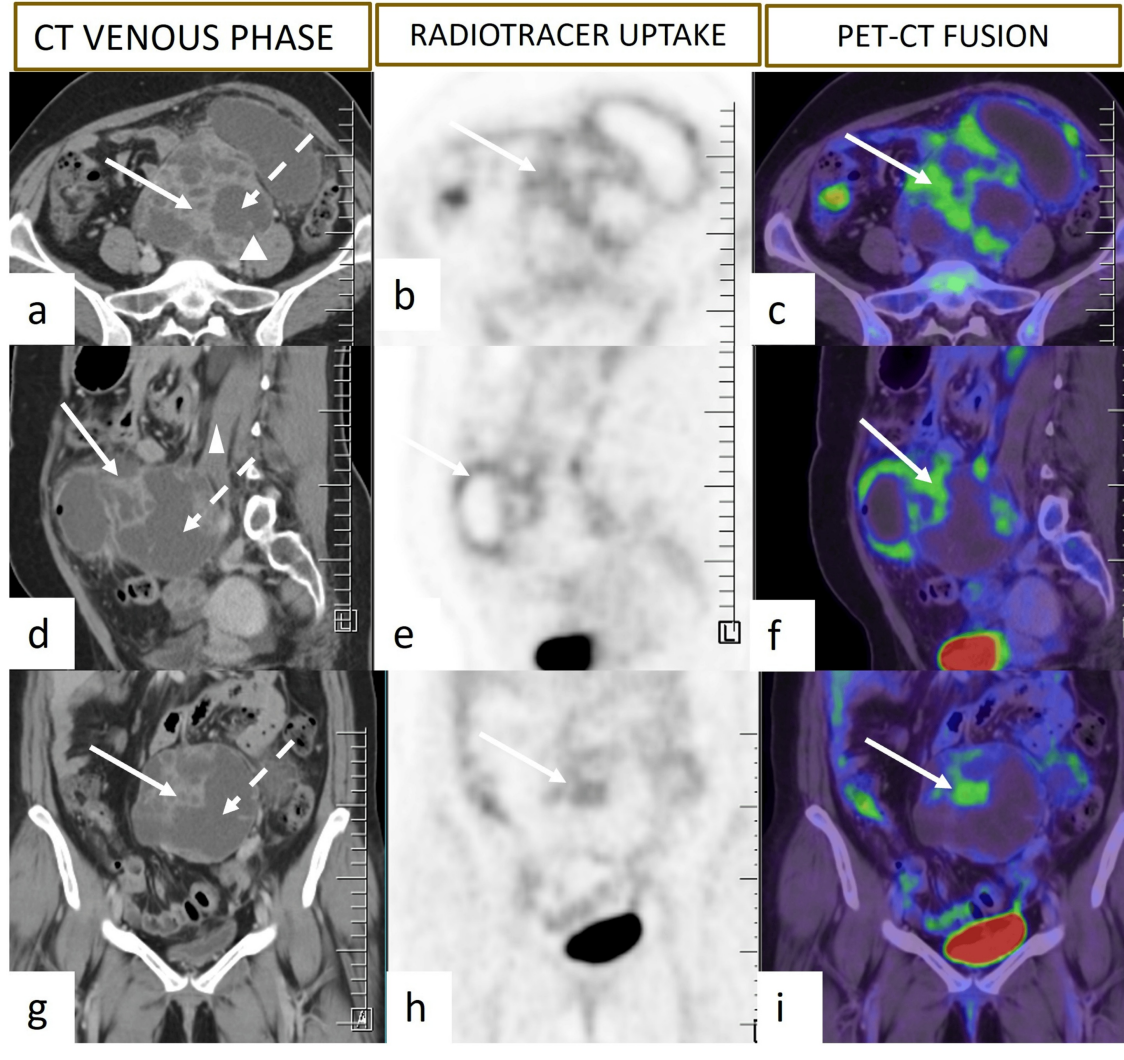
Axial (a, b, and c) and reformatted coronal (d, e, and f) and sagittal (g, h, and i) PET-CT images show a large, FDG-avid (SUVmax: 3.66) multiloculated solid cystic lesion in the mesentery. The solid components are enhancing and FDG-avid (arrow) with central non-enhancing necrotic areas (dashed arrow) within. The lesion is seen abutting the left psoas muscle (arrowhead) and adherent to the sigmoid colon. PET-CT: positron emission tomography-computed tomography, FDG: fluorodeoxyglucose, SUVmax: maximum standardized uptake value

A 34-year-old woman was admitted with insidious onset, gradually increasing lower abdominal pain and distension over a period of 11 months. Initial scans and diagnostic laparoscopy done elsewhere showed an intra-abdominal mass fixed to the sigmoid colon and root of the mesentery. Biopsy was indicative of an endometrial stromal tumor. However, slides reviewed at our center established a diagnosis of gastrointestinal stromal tumor (GIST), and she was initiated on oral imatinib 400 mg/day. Her symptoms continued despite treatment. PET-CT scan revealed a large, FDG-avid (SUVmax: 3.66) multilobulated solid cystic mass in the mesentery with central necrosis and left ureter compression leading to hydroureteronephrosis (Figure 2). The lesion was also fixed to the sigmoid colon and nearby bowel loops.

The patient was taken up for resection of the mesenteric mass, sigmoidoscopy, and right salpingo-oophorectomy on May 17, 2023. Histopathology of the specimen showed small round blue cells with a high nuclear-to-cytoplasmic (N/C) ratio, and IHC established the diagnosis of extraosseous Ewing's sarcoma (CD99 positive and FL1-1 positive). She was pT3N0M0 staged. She was started on adjuvant chemotherapy with a reduced dose of VAC regimen because of previous myelosuppression. During the seventh day after the second cycle, the patient presented with high-grade fever, vomiting, and sore throat. Clinical assessment confirmed febrile neutropenia, with a total white blood cell (WBC) count of 940/μL (reference range: 4,000-11,000/μL) and an absolute neutrophil count (ANC) of 94/μL (reference: >1,500/μL), along with hypotension (blood pressure: 90/60 mmHg).

She was admitted and treated with intravenous fluids, granulocyte colony-stimulating factor (G-CSF), broad-spectrum antibiotics, and supportive therapy. However, her clinical condition worsened, with evidence of multi-organ dysfunction including impaired renal function (serum creatinine: 1.9 mg/dL; reference range: 0.6-1.2 mg/dL), elevated total bilirubin (2.8 mg/dL; reference range: 0.2-1.2 mg/dL), and coagulopathy indicated by an increased international normalized ratio (INR) (1.8; reference range: 0.8-1.2). Despite intensive care measures, including the use of inotropic support and initiation of continuous renal replacement therapy (CRRT), the patient experienced two episodes of cardiac arrest on August 28, 2023, and could not be resuscitated. The terminal cause of death was registered as multi-organ dysfunction due to neutropenic sepsis. This sad case highlights the deadly nature of chemotherapy-induced neutropenia in patients with EES and emphasizes the importance of careful monitoring and dose modification.

Case 3: Huge suprarenal EES posing as adrenocortical carcinoma

**Figure 3:**
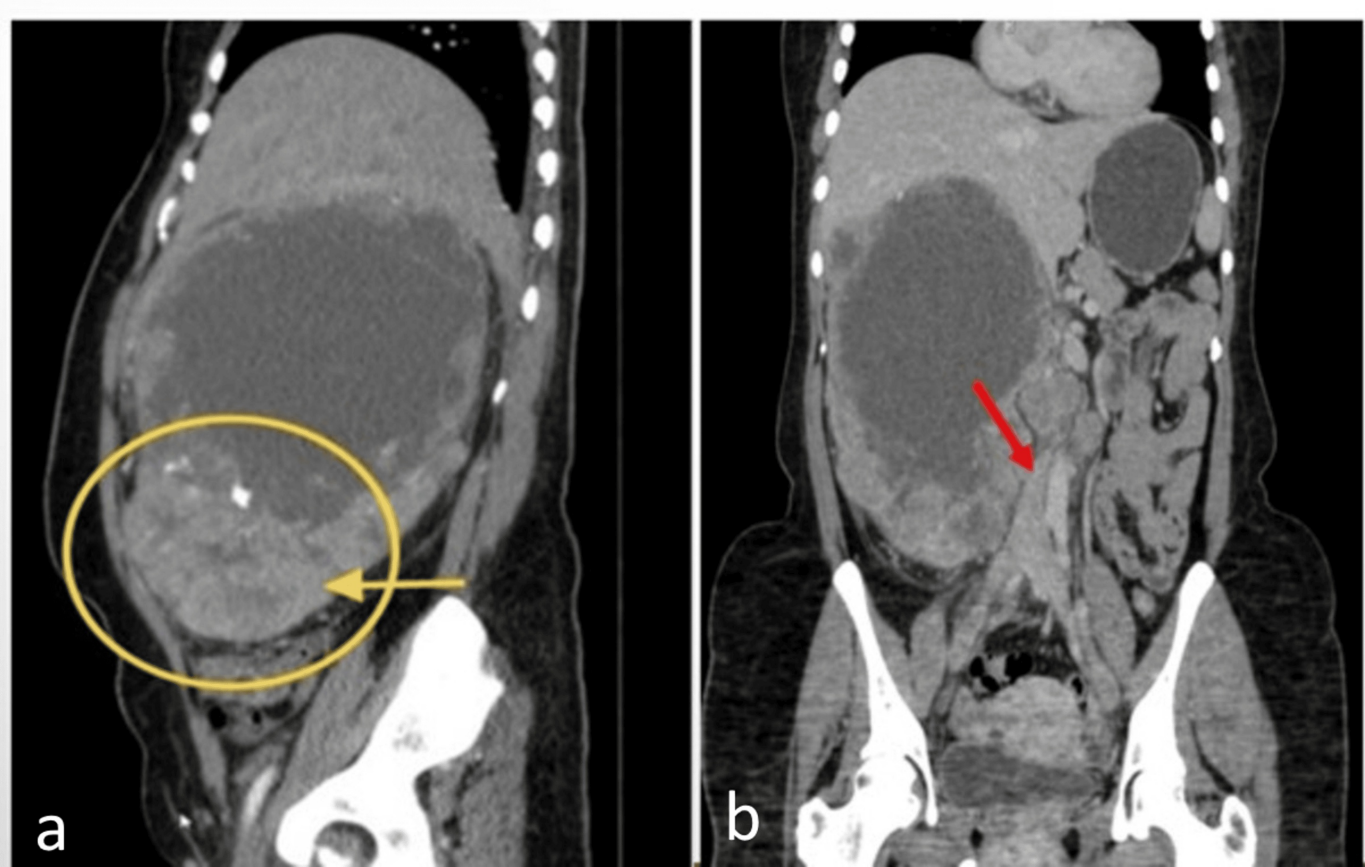
Sagittal CT arterial phase (a) and coronal CT venous phase (b) shows a large heterogeneously enhancing lesion with areas of necrosis. Suspicious reniform structure in the lower pole (yellow circle). Non-visualization of the upper half of the IVC, with the distal half of the IVC seen (red arrow). CT: computed tomography, IVC: inferior vena cava

A 30-year-old woman came with complaints of an upper abdominal mass, constipation, and decreased urine output of one week's duration. She gave a history of insidious onset of swelling in the right hypochondrium and epigastric area with a recent weight loss of 10-12 kg during the last year. On physical examination, there was a large irregular mass, which could be felt 8 cm below the right costal margin. Contrast-enhanced computed tomography (CECT) revealed a large, heterogeneously enhancing soft tissue mass measuring approximately 12.8 × 14.0 × 10.5 cm in the right suprarenal region, with non-enhancing areas suggestive of necrosis (Figure 3). It was compressing the inferior vena cava (IVC) at the hepatic veins' confluence and encasing the infra-diaphragmatic IVC, resulting in severe narrowing.

Additionally, the mass invaded the medial diaphragm, contacted the liver segments V-VIII and caudate lobe, and inferiorly displaced the right kidney. It had a mild supradiaphragmatic extension contacting the pericardium. Differentials given on radiology were adrenocortical carcinoma or sarcomatoid renal tumor. Ultrasonography (USG)-guided Tru-cut biopsy of the mass was done, which on histology showed sheets of small round cells with very little cytoplasm. IHC revealed intense positivity for Ki-67 (70%), CD99, vimentin, and FLI-1, establishing the diagnosis of extraosseous Ewing's sarcoma. Lymphoma, neuroendocrine tumor, and adrenal origin markers (INSM1, S100, inhibin, TTF1, and CD45) were negative.

Endocrine evaluation, including serum cortisol, dehydroepiandrosterone sulfate (DHEAS), and urinary metanephrines, was within normal limits, essentially excluding functioning adrenal tumors. The patient was initiated on neoadjuvant VDC-IE chemotherapy and is being evaluated for surgical resection after tumor shrinkage. This case illustrates the diagnostic dilemma presented by giant retroperitoneal EES masquerading as adrenal neoplasms.

Case 4: Primary infraclavicular EES with sequential multimodal therapy

**Figure 4:**
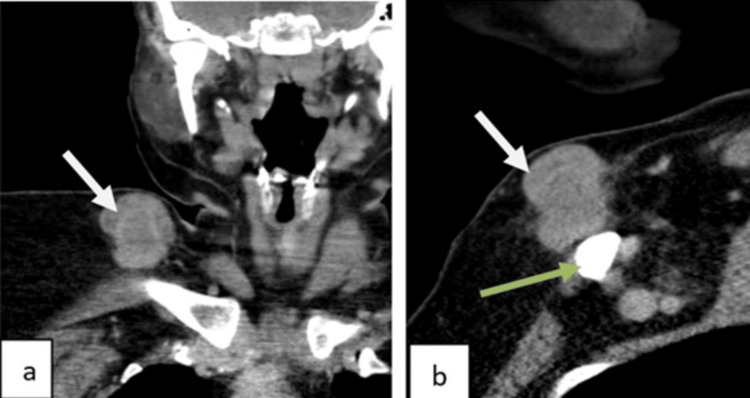
Coronal (a) and sagittal (b) venous phase CT sections show a well-defined heterogeneously enhancing right supraclavicular lesion with areas of necrosis within. No invasion of the right clavicle (green arrow) is noted. Fat planes are maintained with the surrounding structure. CT: computed tomography

A 35-year-old man had a 1.5-year history of a slowly increasing swelling over the right infraclavicular area. On clinical examination, there was a firm, mobile 4 x 3 cm swelling in the subcutaneous plane without any overlying change in the skin or clavicle involvement. PET-CT revealed a sharply defined, FDG-avid mass above the mid-third of the clavicle with a SUVmax of 4, with preservation of fat planes with surrounding structures and no nodal or distant metastasis (Figure 4). CT-guided biopsy consisted of sheets of small round blue cells with increased mitotic activity. IHC established Ewing's sarcoma with CD99, FL1, and NKX2.2 positivity and a Ki-67 index of 40%.

The patient had six cycles of neoadjuvant chemotherapy with VAC-IE regimen and a wide local excision (WLE) done on December 13, 2023. Histopathology demonstrated ypT1Nx with tumor-free margins (nearest: 0.3 cm medial margin) and no lymphovascular invasion. He then received adjuvant external beam radiotherapy (nine fractions) and subsequently came back for resumption of adjuvant chemotherapy. Nevertheless, the disease had evidence of worsening on adjuvant therapy, and a decision was made to start second-line chemotherapy with gemcitabine and docetaxel. The first cycle was tolerated well, and the patient was discharged in stable condition with a directive for close outpatient follow-up. This case demonstrates the need for aggressive multimodal treatment of localized EES as well as the possibility of an early recurrence despite the best initial treatment.

Case 5: Pelvic extraosseous Ewing's sarcoma presenting as prostatic abscess with widespread metastasis

A 35-year-old man presented with difficulty in micturition and a history of previous drainage for suspected prostatic abscess. The symptoms did not resolve with the procedure, and further imaging was ordered. Contrast-enhanced CT of the kidneys, ureters, and bladder (KUB) showed a huge lobulated soft tissue mass lesion replacing the prostate gland, with hypodense areas pointing toward internal necrosis or abscess formation. The mass lesion had a significant mass effect, causing extraneous compression on the urinary bladder.

**Figure 5:**
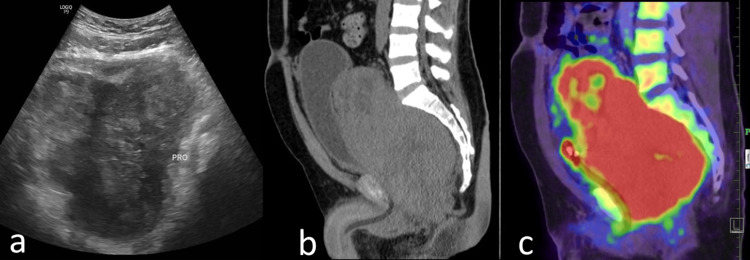
Ultrasound image (a) shows a heterogeneous mass lesion with ill-defined necrotic areas within completely replacing the prostate. The sagittal non-contrast CT (b) shows a large heterogeneous soft tissue mass with central necrotic areas arising from the prostate and infiltrating the urinary bladder anteriorly and rectum posteriorly. This lesion is seen to be intensely FDG-avid on PET-CT scan (c). PET-CT: positron emission tomography-computed tomography, FDG: fluorodeoxyglucose, CT: computed tomography

Ultrasound of the pelvis showed a large, heteroechoic, lobulated mass about the prostate, with hypoechoic foci consistent with central necrosis (Figure 5A). The atypical and aggressive nature of the lesion led to suspicion for a neoplastic process. Ultrasound-guided biopsy was carried out, and histopathology revealed sheets of small blue round cells. Immunohistochemistry led to a diagnosis of extraosseous Ewing's sarcoma (EES).

Additional staging with FDG PET-CT revealed an intensely FDG-avid mass in the pelvis (SUVmax: 16.92), involving and destroying the prostate and encasing the urinary bladder (Figure 5B, 5C). Loss of intervening fat plane between the lesion and rectum was consistent with locally invasive disease. In addition, multiple FDG-avid metastatic deposits were noted in the liver, lungs, and vertebrae, which are indicative of widespread dissemination at presentation.

The diagnosis of EES in this instance was especially difficult because of the initial clinical suspicion of an infective pathology and the unusual pelvic location simulating a urological or prostatic abscess. This case emphasizes the insidious character of EES and the critical contribution of initial imaging, histopathology, and IHC in the diagnosis of soft tissue tumors presenting as imitators of benign urological conditions. It also depicts the aggressive biology and metastatic nature of EES, tending to develop disseminated disease and necessitating urgent systemic treatment.

## Discussion

This case series emphasizes the varied anatomical presentations and diagnostic challenges of EES, a rare and very aggressive Ewing sarcoma family tumor. Our patients had masses in unusual places, such as the mediastinum, mesentery, right suprarenal area, infraclavicular soft tissue, and prostate, consistent with prior observations that EES may occur in nearly every soft tissue, including unusual visceral and retroperitoneal sites [2]. These findings are in concordance with those by Murthy et al., who reported retroperitoneum and chest wall as frequent EES sites in their decade-long experience from a single center in India [4]. In contrast to skeletal Ewing's sarcoma, which predominantly affects adolescents, EES demonstrates a broader age distribution. All our patients were adults, supporting further the literature that EES often presents in adult age groups as opposed to its osseous variant.

The unusual location of the tumor makes its diagnosis difficult, thus delaying treatment. Our case of prostatic Ewing's sarcoma was unique, and to the best of our knowledge, only 10 other cases have been reported to date. Initially, it mimicked as an abscess. However, later, its rapid growth and spread raised the possibility of malignant etiology, and later diagnosed to be EES. Prostatic Ewing's sarcoma, as seen in the literature, has a poor prognosis due to its rapid growth and infiltration into the surrounding pelvic viscera [9].

Histologically, in all our cases, there were small round blue cell tumors with high nuclear-to-cytoplasmic ratios, consistent with classic EES morphology. Immunohistochemistry was essential in making the diagnosis, with membrane positivity for CD99 strongly and markers such as NKX2.2 and FLI-1 further supporting the diagnosis. These observations are in tandem with the reported findings by Shetty et al. in a case of EES in the chin, where initial cytology was deceptive and a precise diagnosis was only obtained through histopathological and immunohistochemical correlation [10]. This underlines the diagnostic difficulty presented by EES, especially when occurring in unusual sites or masquerading as other soft tissue tumors such as lymphoma, rhabdomyosarcoma, or gastrointestinal stromal tumors [1,11].

In one of our patients, who was misdiagnosed with a gastrointestinal stromal tumor at initial presentation, EES was diagnosed only after surgery and IHC. This delay in diagnosis is not unusual but has been well documented in the literature [10], particularly when the tumor arises in the visceral or deep compartments of the anatomy. Early diagnosis is stressed because of the lethal complication seen in one of our patients developing neutropenic sepsis after chemotherapy, a known side effect with aggressive treatment regimens for EES [5].

Therapeutically, the patients were treated with multimodal treatment, regimens of chemotherapy (VAC-IE regimen), surgery, and, in a few instances, radiotherapy. The method is in accordance with current guidelines and replicates therapeutic regimens that have been set up for skeletal Ewing's sarcoma. Chemotherapy is still the cornerstone of treatment, as drugs such as vincristine, doxorubicin, cyclophosphamide, ifosfamide, and etoposide provide significant gains in event-free and overall survival. Grier et al. significantly showed the advantage of combining ifosfamide and etoposide with standard treatment, achieving improved survival in localized disease [6]. In our experience, patients who underwent prompt resection and adjuvant therapy were clinically stable compared with those with late diagnosis or extensive disease at presentation.

Our results also concur with Gaspar et al.'s findings, who stressed that although EES has therapeutic pathways similar to those of skeletal Ewing's sarcoma, treating it is more complicated because of anatomical complexity, greater metastatic tendency, and lack of established clinical guidelines because of its rarity [5]. Additionally, it is also seen that EES, when localized with no distant spread, like our case of infraclavicular tumor, has a better prognosis. However, the aggressive chemoradiotherapy treatment may cause adverse effects in patients with other comorbidities, such as cardiac problems [12].

This case series highlights the clinical heterogeneity, diagnostic traps, and therapeutic dilemmas of EES. Our experience parallels and validates previous reports, further emphasizing the need for a multidisciplinary referral, prompt histopathological and immunohistochemical evaluation, and compliance with aggressive multimodal therapy for a favorable prognosis.

## Conclusions

Extraosseous Ewing's sarcoma continues to be an uncommon and diagnostically troublesome cancer with extremely variable anatomical presentations, which tend to masquerade as other soft tissue tumors and provoke delayed or incorrect diagnosis. Our series of cases reaffirms the absolute need for a heightened index of suspicion in adults with unusual soft tissue masses, especially if they are deep-seated or visceral. Histopathology complemented by immunohistochemistry, most notably by markers such as CD99, NKX2.2, and FLI-1, is still fundamental to correct diagnosis. Early diagnosis, staging according to standards, and implementation of a multidisciplinary, multimodal therapeutic plan including surgery, chemotherapy, and radiotherapy are essential to enhancing clinical outcomes. Despite advances in therapeutic strategies, prognosis remains guarded in patients with advanced disease, underscoring the need for early intervention and close monitoring. Larger multicentric studies and registries are needed to better define prognostic markers and to develop standardized treatment protocols tailored to this aggressive and enigmatic tumor.
